# Supplementary material for: Epithelial–Macrophage Crosstalk in Host Responses to Campylobacter jejuni Infection in Humans
Source: Microorganisms. 2025 Dec 10;13(12):2808. doi: 10.3390/microorganisms13122808 (PMC12735976; doi:10.3390/microorganisms13122808)
Supplement: Supplementary file 1 [file microorganisms-13-02808-s001.zip › microorganisms-4030473-supplementary.pdf]

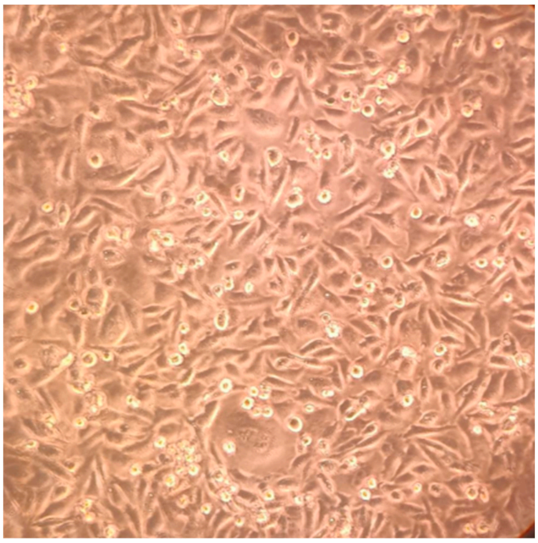

Supplementary Figure S1 shows the adherence of HT-29 cells after 4 hours of incubation.

Supplementary Table S1: Raw data of the gentamycin protection assay.

Group	Replicates	T0	T2	Killing percentage ((T0-T2)/T0) x 100
		CFU/ml	CFU/ml	
10 MOI	1	14200	2400	83.09859
	2	17400	2500	85.63218
	3	3760	2240	40.42553
	4	22400	1900	91.51786
	5	15600	1520	90.25641
50 MOI	1	16600	1540	90.72289
	2	14200	2120	85.07042
	3	3620	3300	8.839779
	4	18000	3740	79.22222
	5	15800	1900	87.97468
Negative control	1	1980	2520	
	2	3140	1400	55.41401
	3	3380	1740	48.52071
	4	3420	2340	31.57895
	5	18600	3600	80.64516
